# Supplementary material for: Experimental animal models and their use in understanding cysticercosis: A systematic review
Source: PLoS One. 2022 Jul 19;17(7):e0271232. doi: 10.1371/journal.pone.0271232 (PMC9295976; doi:10.1371/journal.pone.0271232)
Supplement: Attachment — Submitted filename: Review PONE-D-22-01651.pdf [file pone.0271232.s002.pdf]

Dear Editor,

Here is my comment on the manuscript PONE-D-22-01651, "Experimental animal models and their use in understanding cysticercosis: A systematic review."

It is an interesting article where a systematic bibliographical review is carried out, of the different animal models that have been developed to study the interaction between Taenia spp. and its intermediate host. It should be accepted with some major changes

My biggest criticism is in the section on the strengths and weaknesses of the animal models, should be highlighted as a tool to understand the pathogenesis of neurocysticercosis. As the reproducibility of the animal model, the clinical picture and pathology that develops. As for example, the use of pigs to study NCC has the advantage of being the natural host, however it is difficult to have a good reproducibility of the experimental infection of the central nervous system, while the use of rats presents a high reproducibility, but has the disadvantage of not being the natural host. The use of immunosuppressed animals has many limitations to understand the host-parasite interaction. To have animal model with characteristic of clinical symptoms and pathology similar to human , it is help to study the disease.

My minor criticism is in Table 1, the study by Alan Mejia Maza (2018) should be included, where they use the NCC rat model, in which they compare two routes of infection (intracranial and oral).

On the other hand, in Table 1, in the published article by Verastegui et al. (2015), the number of cysts obtained in rats with NCC is reported.

Sincerely

Manuela Verástegui P.
